# GoAstV Induced Mitophagy and Ferroptosis in the Spleen of Goslings

**DOI:** 10.3390/ani16193019

**Published:** 2026-09-25

**Authors:** Yunfeng Chen, Duanfeng Fu, Haiqin Li, Yan Shi, Zhihua Lu, Haoyu Huang, Xiaona Gao, Xiaoquan Guo

**Affiliations:** 1 Jiangxi Provincial Key Laboratory for Animal Health, College of Animal Science and Technology, Jiangxi Agricultural University, Nanchang 330045, China; cyf13579@stu.jxau.edu.cn (Y.C.); 15350315392@163.com (D.F.); 15170035002@163.com (Z.L.); icehaoyu@foxmail.com (H.H.); xiaona.gao@jxau.edu.cn (X.G.); 2 Institute of Animal Husbandry and Veterinary Medicine, Jiangxi Academy of Agricultural Sciences, Nanchang 330200, China; lihaiqin522@126.com; 3 School of Computer and Information Engineering, Jiangxi Agricultural University, Nanchang 330045, China; shiyan0015@126.com

**Keywords:** GoAstV, mitophagy, ferroptosis, spleen, goslings

## Abstract

A novel goose astrovirus (GoAstV) has emerged in various provinces of China, leading to a mortality rate of up to 50% among GoAstV goslings. The spleen is an important immune organ in geese. Studies have shown that goslings infected with GoAstV exhibit higher viral loads in their spleens, and the spleens sustain damage. However, the mechanism underlying its induction of spleen injury remains to be elucidated. Therefore, a gosling infection model with GoAstV was established in this experiment. The Con group was subcutaneously injected with 0.2 mL of saline, while the GoAstV group was subcutaneously injected with GoAstV JX01 (2 × 10^6^ TCID50/0.2 mL). Spleen tissues were collected on days 1, 3, 6, 9 post-infection (dpi). The results showed that the body weight of the GoAstV group decreased significantly, while the spleen weight and spleen index increased significantly. Post-mortem examination revealed that the spleen of the GoAstV group was significantly enlarged. The viral load in the spleen reached its peak at 6 dpi. Histopathological and electron microscopy revealed lymphocyte necrosis, autophagic lysosomes, and mitochondria autophagosomes. The spleen of the GoAstV group exhibited increased iron content, elevated malondialdehyde (MDA) production, decreased glutathione (GSH) content, and reduced glutathione peroxidase (GSH-Px) activity. RT-qPCR and Western blot analyses revealed that GoAstV upregulated mRNA expression of pro-inflammatory cytokines and inhibited anti-inflammatory cytokines. GoAstV also upregulated genes and proteins linked to ferroptosis and mitophagy, while downregulating those associated with anti-ferroptosis and SQSTM1. In conclusion, our findings suggest that GoAstV infection may induce mitophagy and ferroptosis in the gosling spleen, thereby contributing to splenic injury.

## Simple Summary

A novel goose astrovirus has emerged in China, causing up to 50% mortality in young geese. Since the spleen is a vital immune organ and is heavily damaged during infection, we aimed to understand how this virus injures it. We infected goslings with the virus and examined their spleens over nine days. We found that infected birds lost weight while their spleens became abnormally enlarged. Under the microscope, we observed significant cell death in the spleen tissue. Further analysis revealed that the virus triggers strong inflammation and activates two harmful processes: iron-driven cell death and self-destruction of energy-producing structures within cells. These findings help explain why this virus is so deadly to young geese. Our study provides a clearer understanding of the disease mechanism, which could guide the development of new treatments or preventive strategies to protect the poultry industry.

## 1. Introduction

Goose astrovirus (GoAstV), a member of the family Astroviridae, is recognized as the primary etiological agent responsible for acute gout and systemic urate deposition in infected goslings [1,2]. Since the first report of GoAstV in 2017, outbreaks of this disease have occurred in major goose farming regions of China, resulting in significant economic losses to the poultry industry [3,4]. GoAstV exhibits broad tissue tropism and can replicate in various organs [5]. The spleen, being the largest and most efficient immune organ, serves as the central site for the occurrence of cellular and humoral immunity, playing roles in immune surveillance, regulation, and filtration. Studies have reported high viral loads in the spleens of goslings with GoAstV, with observations of lymphocyte apoptosis, reticular fiber destruction, and decreased levels of CD8 T cells in the splenic tissue [6]. However, the underlying mechanism by which GoAstV infection induces spleen injury in goslings has not been fully elucidated.

Mitochondria, as crucial organelles within cells, play an irreplaceable role in multiple key physiological processes. Mitochondria are involved in a dynamic equilibrium regulation process, primarily through key processes such as mitochondrial fission, mitochondrial fusion, and mitochondrial autophagy [7,8]. Studies have shown that when stimulated by pathological or external conditions, this regulation process is prone to dysfunction. Mitophagy serves as a selective way to isolate and degrade damaged or incomplete mitochondria, contributing to the maintenance of mitochondrial network integrity and cellular homeostasis [8]. Autophagy is originally a potent host cell defense mechanism against viral infections. However, through continuous evolution, viruses have acquired a strong ability to hijack and subvert autophagy for their benefit [9]. Autophagy plays a dual role in viral infections, as it can both facilitate the clearance of previral components and promote the elimination of mitophagy, thereby leading to intricate immune evasion mechanisms [10]. However, there is currently no related report on whether mitophagy occurs in the spleen of goslings after GoAstV infection.

Additionally, it is noteworthy that mitochondria are also the primary sites for iron utilization and oxidative metabolism, as well as the main sources of reactive oxygen species (ROS). Damage and impairment of mitochondrial function can promote oxidative stress, leading to the induction of ferroptosis. The relationship between ferroptosis and mitochondrial function is receiving increasing attention. Research indicates that the hemagglutinin of influenza A virus induces ferroptosis, facilitating viral replication [11]. However, whether ferroptosis occurs in the spleen of goslings after GoAstV infection remains to be further investigated.

Ferroptosis represents a distinct modality of regulated cell death that is fundamentally driven by iron-dependent mechanisms, characterized by iron accumulation, lipid peroxidation, and antioxidant system imbalance. Iron is an essential nutrient involved in numerous fundamental biological processes, including mitochondrial respiration, gene regulation, and DNA synthesis or repair [12]. However, excessive iron is associated with biotoxicity, disrupting redox homeostasis, inducing oxidative stress, causing the accumulation of lipid ROS, and ultimately leading to oxidative cell death [13].

While substantial efforts have been directed toward the isolation, molecular characterization, and prevention of Goose astrovirus (GoAstV), the specific pathogenic mechanisms driving splenic injury during infection remain largely unexplored. This study focuses on the dynamic changes and interrelationships of mitophagy- and ferroptosis-associated molecules in the spleen following GoAstV infection, aiming to explore the potential mechanisms underlying splenic injury in geese. This research contributes to a deeper understanding of the pathogenic mechanisms and provides theoretical support for disease prevention and control.

## 2. Materials and Methods

### 2.1. Virus

The GoAstV-2 JX01 strain used for animal experiments in this study was obtained from the Institute of Animal Husbandry and Veterinary Medicine, Jiangxi Academy of Agricultural Sciences, and its corresponding accession number is MZ576222.1. It is stored in a −80 °C ultra-low temperature freezer in the laboratory.

### 2.2. Experimental Design

Two hundred one-day-old Xingguo gray geese were purchased from a poultry farm in Jiangxi Province and randomly assigned to control or GoAstV-infected groups *(n* = 100/group). The groups were housed separately under comparable environmental conditions. Eight goslings per group were euthanized at each of four time points, yielding 32 scheduled samples per group. The remaining animals were reserved to compensate for potential post-challenge losses and were excluded from statistical analyses. Overall mortality was 5%. According to previous modeling methods, Virus challenge tests were conducted on the same day, with each goose in the infection group inoculated subcutaneously with GoAstV-2 JX01 (2 × 10^6^ TCID50/0.2 mL), while each goose in the control group received subcutaneous injection of 0.2 mL sterile physiological saline [1,14,15,16]. Prior to challenge, randomly selected geese were tested for GoAstV, and no viral load was detected, with negative results [1]. At 1, 3, 6, and 9 days post-infection (dpi), 8 goslings from each group were randomly selected, euthanized by intravenous injection of pentobarbital sodium under sterile conditions, and their spleens were quickly weighed and photographed. They were then rapidly weighed and photographs of their spleens were taken. Some spleen samples were collected in 2 mL cryogenic vials and stored in a −80 °C freezer; other samples were fixed in sectioning solution for pathological observation of spleen tissue and in electron microscopy fixative for ultrastructural analysis of spleen cells.

### 2.3. Pathology of Organizations

The spleen tissue was fixed using a 4% paraformaldehyde solution, followed by rinsing, ethanol dehydration, transparency, and embedding in paraffin wax. Sections were obtained, stained with hematoxylin and eosin, then observed under an optical microscope (Leica Microsystems, Wetzlar, Germany) to examine the histopathological changes in the spleen tissue.

### 2.4. Transmission Electron Microscopy

The spleen tissue samples fixed in a 2.5% glutaraldehyde solution were rinsed with phosphate buffer, followed by fixation in a 1% osmic acid solution and subsequent rinsing. Dehydration was carried out using varying concentrations of ethanol, followed by processing with acetone and embedding solution, then heated to obtain the final product. The samples were sectioned using an ultrathin microtome and double stained with 3% uranyl acetate and lead citrate solutions. The sample was sectioned with an ultramicrotome and then double-stained with 3% uranyl acetate solution and lead citrate solution. Finally, observation was conducted under a transmission electron microscope.

### 2.5. Determination of Viral Load in the Spleen

The quantification procedure of GoAstV load in the spleen is established upon experimental methodologies outlined in prior research [17]. Extract total RNA from spleen samples of the GoAstV group, synthesize primers and probes, establish a standard curve according to the relevant conditions provided in the article, and determine the viral load in the spleen. The detection limit of GoAstV-2 by qPCR is 10 copies/μL, and there are no false positives for other common avian influenza viruses.

### 2.6. Determination of Oxidation, Antioxidant Indexes, and Tissue Iron Content in the Spleen

Following the pre-treatment of spleen tissue samples as instructed in the reagent kit manual (Jiancheng Bioengineering Institute, Nanjing, China), the measurement of glutathione (GSH), glutathione peroxidase (GSH-Px), malondialdehyde (MDA), lipid peroxidation (LPO), and iron content was conducted strictly in accordance with the subsequent operational steps outlined in the manual.

### 2.7. Total RNA Extraction and RT-qPCR Analysis

Total RNA was extracted from the spleen tissues of experimental animals using the TRIzol method (Yeasen, Shanghai, China). The extracted RNA was then reverse-transcribed into cDNA, and the resulting cDNA was stored at −20 °C for subsequent RT-qPCR analysis. The qPCR reaction program and system were strictly followed as described in the reagent manual (Vazyme, Nanjing, China). Primer 5.0 was utilized for the design of fluorescent quantitative PCR primers, as outlined in Table 1. Data obtained from real-time fluorescent quantitative PCR amplification were analyzed using the delta threshold cycle method for comparison of GAPDH levels, normalized to calculate relative expression levels, and visualized using GraphPad Prism (version 9.3.0, GraphPad Software, San Diego, CA, USA) for experimental data presentation.

### 2.8. Western Blot

The RIPA reagent was employed along with proteinase inhibitors and phosphatase inhibitors (Solarbio, Beijing, China) for protein extraction from spleen tissue following standard procedures.

The total protein content of the tissue was determined using a BCA kit (Solarbio, Ltd., Beijing, China), and the protein concentration of each sample was adjusted to 5 mg/mL. Proteins of different sizes were separated by SDS-PAGE, and then transferred from the gel to a PVDF (0.45 μm) (Sigma-Aldrich, Darmstadt, Germany) membrane and blocked. Immunoblotting was then carried out with antibodies. Protein signals were visualized using the Bio-Rad Chemidoc Touch imaging system (Bio-Rad, Hercules, CA, USA), and quantitative analysis of the respective protein grayscale values was performed using Image J (ImageJ-win64, NIH, Bethesda, MD, USA) software. Anti-LC3 primary antibody was purchased from Abmart (Shanghai, China), anti-GAPDH antibody from Sigma-Aldrich (St. Louis, MO, USA), and all other antibodies from Wanleibio Co., Ltd. (Shenyang, China).

### 2.9. Immunohistochemistry

After slicing the spleen paraffin blocks, subjected to dewaxing, hydration, and antigen retrieval, followed by treatment with 3% H_2_O_2_ and blocking with 5% bovine serum albumin for 1 h. Sections were incubated overnight at 4 °C with a mouse monoclonal anti-TRF primary antibody (Abmart, Cat.T56618, 1:200 dilution), followed by incubation with HRP-conjugated goat anti-mouse IgG secondary antibody (Proteintech, Wuhan, China, 1:500 dilution) for 30 min at room temperature. Signal was visualized using DAB chromogen. The analysis was conducted using the ImageJ software. Briefly, a consistent threshold was applied across each experimental batch to isolate DAB-positive signals, and quantitative indicators of TRF expression and mean optical density were recorded from five randomly selected, non-overlapping ×400 fields per section in a blinded manner.

### 2.10. Statistical Analysis

All data are presented as the margin of error (M ± SE). The independent sample T test was used to compare the differences between the two groups. One-way analysis of variance (ANOVA) was used to compare the differences between multiple groups. The experimental data conformed to a normal distribution; GraphPad Prism software (version 9.3.0) was used for visualization and plotting. Statistically significant threshold, respectively (*p* < 0.05) (*), *p* < 0.01 (**), *p* < 0.001 (***), if *p* < 0.05, significant difference.

## 3. Results

### 3.1. Phenotypic Effects of GoAstV Infection in Goslings

As shown in Figure 1, significant changes in body weight and spleen phenotype were observed in goslings following GoAstV infection. Compared with the control group, goslings infected with GoAstV showed a significant decrease in body weight at both 6 days and 9 days (Figure 1B). At the same time, spleen weight and spleen index increased significantly at 3, 6, and 9 dpi (Figure 1C,D). Additionally, the anatomical image at 6 dpi revealed a noticeable enlargement of the spleen in the GoAstV group. Viral load measurement in the spleen indicated the presence of viral copies at 1 dpi, with the copy number peaking at 6 dpi.

### 3.2. GoAstV Altered the Organization and Ultrastructure of the Gosling Spleen

Subsequently, we observed the histopathological and ultrastructural changes in the spleen tissues of goslings infected with GoAstV. H&E staining revealed intact splenic architecture with distinct red and white pulp boundaries in the control group. Conversely, GoAstV infection caused indistinct red-white pulp boundaries and lymphocyte necrosis (Figure 2A). Additionally, ultrastructural observations revealed that the spleen cells in the control group had an intact morphological structure, with clear mitochondrial structure and no significant abnormalities. However, the mitochondrial structure within the cells was indistinct, with increased membrane fragmentation, loss of cristae, and the presence of autolysosomes and mitophagosomes in the GoAstV group (Figure 2B).

### 3.3. GoAstV-Induced Inflammatory Response in the Spleen of Goslings

As the spleen is a vital immune organ, this study investigated changes in the mRNA expression of inflammation-related genes in the spleen of goslings following GoAstV infection. The results showed that compared to the control group, the GoAstV group exhibited an upward trend in the levels of inflammatory cytokines IL6, IL2, TNF-α, and IFN-γ, while the anti-inflammatory cytokine IL10 showed a downward trend at 3 dpi and 6 dpi (Figure 3). This indicated that GoAstV infection activates the immune function through pro-inflammatory cytokines and suppresses the expression of anti-inflammatory cytokines in the gosling spleen, also indirectly suggesting that the spleen is damaged. Figure 3. Changes in inflammatory-related molecules in the spleen of GoAst-infected goslings. Expression levels of mRNA of Splenic inflammatory factors IFN-γ (A), IL-10 (B), IL-2 (C), TNF-α (D), and IL-6 (E). F. The heatmap illustrates the expression levels of genes associated with inflammatory factors. Con (control group); Dis (GoAstV group). All data are expressed as M ± SE and analyzed using independent samples *t*-test. *p* < 0.05 (*). *p* < 0.01 (**).

### 3.4. GoAstV-Induced Mitophagy in the Spleen of Goslings

To investigate whether mitochondrial autophagy occurs in the spleen of goslings after GoAstV infection, we measured the expression levels of mRNA and proteins related to mitochondrial autophagy in spleen tissues. The results showed that, compared to the control group, the mRNA expression levels of BECN1, PINK, PRKN, LC3A, and LC3B in the GoAstV group were significantly increased at 3 and 6 dpi (Figure 4A–C,E,F), while the mRNA level of SQSTM1 was decreased (Figure 4D). Meanwhile, the protein expression levels showed significant changes at 9 dpi (Figure 4G,I–L).

### 3.5. GoAstV-Induced Iron Accumulation in the Spleen of Goslings

Iron plays a crucial role in maintaining mitochondrial function. Therefore, to investigate the effect of GoAstV infection on the iron metabolism pathway in the spleen of chicks, we further measured the iron content in spleen tissues as well as the gene and protein expression levels of key molecules in the iron metabolism pathway.

Compared to controls, splenic iron content in the GoAstV group significantly increased at 3 and 6 dpi (Figure 5A). qPCR analysis showed upregulated TRF, TFRC, and NCOA4 (peaking at 6 dpi), concomitant with downregulated FTH1 and FTL at 3 and 6 dpi; all returned to baseline subsequently (Figure 5B–F). These transcriptional trends were corroborated by Western blotting (Figure 5H–K) and increased TF protein expression via immunohistochemistry (Figure 5L,M). Collectively, these data indicate that GoAstV induces splenic iron accumulation.

### 3.6. GoAstV Induced Lipid Peroxidation and Ultimately Led to Ferroptosis

Imbalance in intracellular iron homeostasis leads to an increase in divalent iron ions within the cell, resulting in the generation of toxic ROS substances mediated by iron ions. These substances combine with intracellular lipids to form lipid peroxides that attack the cell bio-membrane, ultimately inducing ferroptosis [18]. To investigate whether GoAstV infection induces ferroptosis in gosling spleen cells, we assessed the changes in the levels of T-GSH, GSH-Px, LPO, and MDA in spleen tissues. The results showed that compared with the control group, the levels of T-GSH and GSH-Px in the GoAstV group significantly decreased at 3 dpi and 6 dpi, while the MDA level significantly increased compared to the control group; LPO significantly increased at 6 dpi (Figure 6A–D). In addition, qPCR results showed that, compared with the control group, the mRNA levels of SLC7A11 and GPX4 decreased at 3 dpi and 6 dpi, while the mRNA levels of ACSL4, LPCAT3, and PTGS2 increased at 3 dpi and 6 dpi (Figure 6E). The WB results showed that the protein expression levels of ACSL4, LPCAT3, and PTGS2 were generally consistent with the gene levels, and exhibited significant changes at 6 dpi (Figure 6F,H). Our findings suggest that GoAstV infection is associated with impaired antioxidant function and activation of the lipid peroxidation pathway in the gosling spleen, which may collectively contribute to the induction of ferroptosis.

### 3.7. Correlation Analysis of GoAstV Infection-Induced Splenic Mitophagy and Ferroptosis

To investigate the relationship between GoAstV-induced mitophagy and ferroptosis in the spleen, we conducted a correlation analysis between the two using the Mantel test. The results showed that the expression of autophagy and mitophagy genes was positively correlated with that of pro-ferroptosis genes at a high level. Meanwhile, the spleen function index was highly correlated with the expression of mitophagy-related genes such as FTL, PRKN, PINK, LC3B, as well as pro-ferroptosis-related genes such as ACSL4, LPCAT3, PTGS, NCOA4 (Figure 7).

## 4. Discussion

GoAstV has emerged in various regions of China, inducing huge economic losses for the goose industry [19]. Recent studies have shown that GoAstV infection can induce spleen damage, activate the host’s innate and adaptive immune responses, lead to lymphocyte apoptosis, reticular fiber destruction, and a reduction in CD8 T cell levels, which is considered one of the important reasons why goslings are more susceptible to GoAstV [6,20]. Although there are studies reporting that GoAstV induces spleen damage, the exact mechanism of this damage remains unclear. Therefore, this study aimed to explore the pathogenic mechanism of GoAstV in the spleen of goslings.

In this experiment, the GoAstV-2 strain was used to infect goslings to establish the infection model, and spleen tissues were collected for subsequent detection. Currently, GoAstV-2 is the primary pathogen causing gout in geese, exhibiting stronger infectivity and higher mortality rates [21]. Epidemiological survey results indicated that GoAstV-2 was more widely prevalent than GoAstV-1 in China [22]. During the challenge experiment, it was observed that goslings began to exhibit clinical symptoms on the third day post-challenge, and a significant decrease in body weight was noted at 6 dpi. Necropsy results revealed significant splenomegaly in the infected goslings, indicating that the spleen phenotype had changed following GoAstV infection. This finding is consistent with the results reported by Ding R et al. [6]. Furthermore, H&E staining and ultrastructural observation of the spleen tissue demonstrated that the spleen tissue was damaged after GoAstV infection. The timing of this phenotypic change coincided with the peak viral load in the spleen. In conclusion, GoAstV infection can cause spleen damage in goslings.

Mitophagy is closely associated with various physiological and pathological states of organisms [23]. Our results suggest that GoAstV infection may trigger mitophagy and ferroptosis in the gosling spleen. While we did not directly assess viral replication or immune evasion in this study, it is plausible that these processes may facilitate viral persistence. Previous studies have shown that viruses such as influenza A virus and Newcastle disease virus can exploit mitophagy to degrade mitochondrial antiviral-signaling protein (MAVS), thereby dampening the host innate immune response [10]. In general, viral infection of host cells typically induces autophagy, a cellular process that facilitates pathogen clearance and triggers the host immune response [24]. However, certain viruses have evolved strategies to not only evade autophagic elimination but also exploit the characteristics of autophagic cell invasion to regulate mitophagy through diverse molecular mechanism hijacking. The hijacking of mitophagy by viruses serves to suppress the host innate immune response—including the inhibition of apoptosis, interferon (IFN) signaling, and inflammasome activation—thereby promoting viral replication and acting as a “safe haven” for viral particles to escape immune surveillance by the host organism [25]. Therefore, we speculate that the observed mitophagy may represent a potential mechanism by which NIBV modulates the host environment, although further studies are needed to confirm this hypothesis.” Therefore, we also detected the level of inflammation in the spleen. The results showed that the mRNA expression levels of pro-inflammatory factor-related genes (IL2, IL6, TNF-α and IFN-γ) increased and the mRNA expression level of anti-inflammatory factor-related gene (IL-10) decreased. This indicates that after GoAstV invaded the spleen, it raised the level of inflammatory factors and did not suppress the inflammatory response.

Iron is an essential trace element in the human body and plays a significant role in the competition between viruses and hosts, being considered a key factor in ferroptosis [26]. It has been reported that viral infections are often accompanied by disturbances in iron ion concentrations [22]. Research by Gong et al. indicated that the Newcastle disease virus (NDV) promoted viral replication by triggering autophagy [27]. Our study showed that GoAstV infection affects iron metabolism in the spleen, manifested by increased iron uptake in the spleen and the occurrence of ferritinophagy. Previous studies have demonstrated that, in antioxidant metabolism, viruses enhance viral proliferation by inhibiting the System Xc-/GPX4 axis. Depletion of GSH leads to the inactivation of GPX4, thereby inducing ferroptosis due to the accumulation of lipid radicals [27]. In our study, GoAstV infection affected the glutathione-related antioxidant function in the spleen of goslings, mainly manifested by a decrease in T-GSH content, a reduction in GSH-Px activity, a decline in the mRNA expression levels of SLC7A11 and GPX4, and the inhibition of GPX4 protein expression. Lipid peroxidation is a hallmark of ferroptosis, with the Fenton reaction and iron-dependent enzymatic reactions directly catalyzing the formation of lipid radicals. This experiment found that GoAstV infection increased the content of MDA and LPO in the spleen, upregulated the mRNA expression levels of ACSL4, LPCAT3, and PTGS2, and increased the expression of ACSL4 and PTGS2 by measuring relevant oxidative indicators. In summary, the above findings suggest that GoAstV infection induces iron accumulation in the spleen tissue of goslings, activates lipid metabolism pathways, inhibits antioxidant functions, and ultimately leads to ferroptosis in spleen cells. In this study, we observed significant alterations in key ferroptosis markers following NIBV infection, including GSH depletion, GPX4 downregulation, and elevated levels of lipid peroxidation products (MDA and LPO), along with upregulation of ACSL4. These findings are consistent with the biochemical hallmarks of ferroptosis and suggest that NIBV-induced renal injury may involve a ferroptosis-like cell death pathway. However, we acknowledge that direct pharmacological rescue experiments using ferroptosis-specific inhibitors (e.g., Fer-1 or DFO) were not included in the present study due to current experimental constraints. While our multi-dimensional evidence aligns with established diagnostic criteria for ferroptosis, future studies incorporating functional validation through inhibitor rescue assays (e.g., 3-MA, Mdivi-1) will be necessary to further confirm the causal role of ferroptosis in NIBV-associated kidney damage and to elucidate the underlying molecular mechanisms.

Recent studies have gradually revealed significant interactions between ferroptosis and autophagy, indicating that ferroptosis requires autophagy mechanisms to execute, particularly selective autophagy, which plays a crucial role in driving cells toward ferroptosis [28]. In response to mild stress or during the early stages of iron overload, mitophagy may sequester iron within mitochondria, thereby reducing the source of ROS in ferroptosis. However, excessive mitophagy may eventually provide additional iron, amplifying lipid peroxidation and ferroptosis [29]. Therefore, the extent of autophagic flux in this process may vary, and its impact on ferroptosis may also differ. Correlation analysis revealed a strong association between mitophagy and ferroptosis markers, suggesting a potential interplay between these two pathways. However, the specific mechanisms linking mitophagy and ferroptosis in the spleen of GoAstV-infected goslings remain to be further elucidated.

It should be noted that the spleen is a highly heterogeneous organ composed of diverse immune and stromal cell populations. Although the present study did not isolate specific cell subpopulations due to technical constraints, accumulating evidence suggests that splenic macrophages and T lymphocytes are key players in inflammatory responses and oxidative stress under viral infection conditions [30]. Macrophages, in particular, are highly susceptible to ferroptosis due to their iron-rich phagocytic nature and abundant polyunsaturated fatty acids in their membranes, which serve as substrates for lipid peroxidation [31]. Similarly, mitophagy has been reported to be activated in macrophages and dendritic cells during viral infections to maintain mitochondrial homeostasis and regulate inflammatory signaling [32]. Therefore, we speculate that the mitophagy and ferroptosis observed in this study may predominantly occur in splenic macrophages and/or T cells. Future studies utilizing single-cell RNA sequencing or flow cytometry with chicken-specific markers are warranted to precisely map these cellular events and dissect the cell-type-specific mechanisms underlying GoAstV-induced splenic injury.

## 5. Conclusions

GoAstV infection results in reduced growth performance, splenic damage, and inflammation in goslings. Our findings suggest that GoAstV-induced splenic damage may be mediated by the induction of autophagy and mitophagy, activation of the lipid peroxidation pathway, accumulation of iron and lipid peroxides, and depletion of GSH, ultimately triggering ferroptosis. This study elucidates the potential mechanisms underlying splenic injury in GoAstV infection, offering novel insights for the prevention of GoAstV and the development of related therapeutic agents.

## Figures and Tables

**Figure 1:**
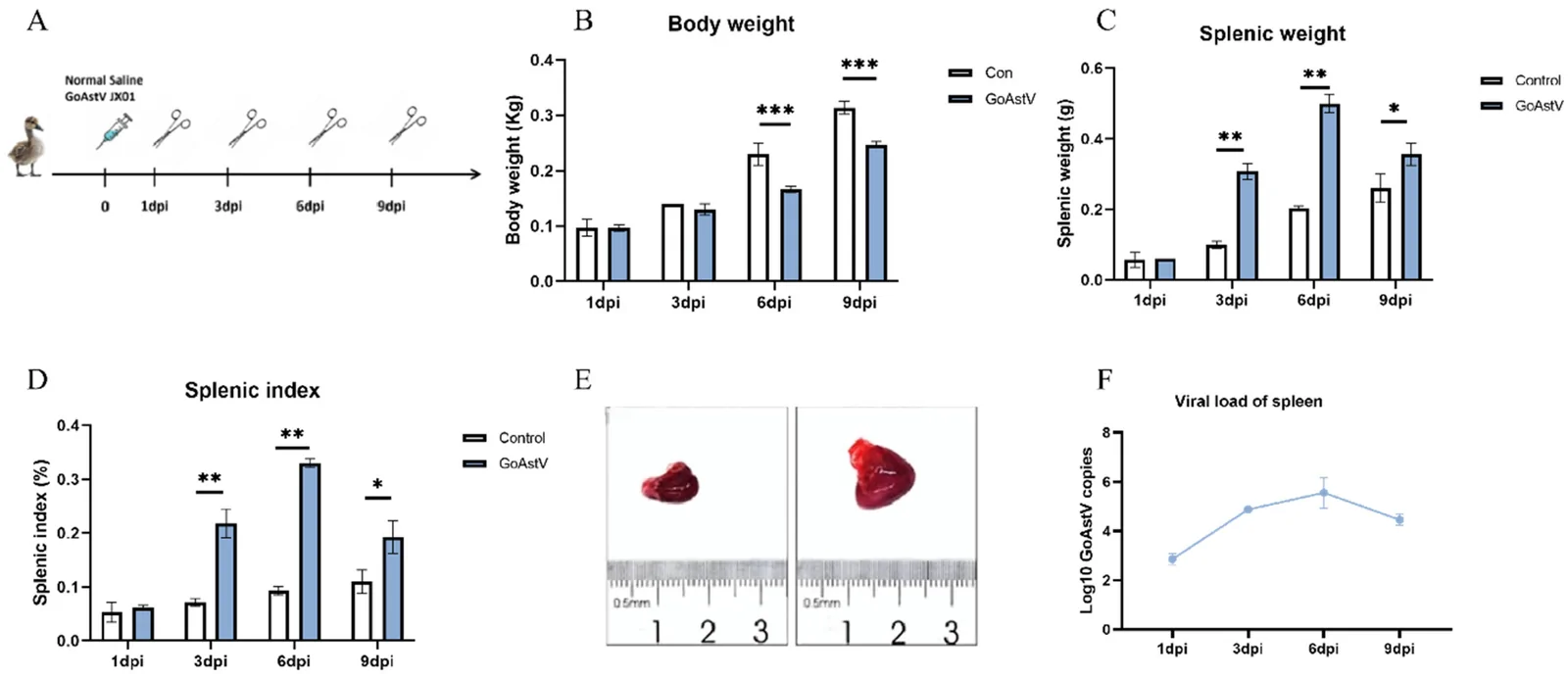
Changes in apparent indices in GoAstV-infected goslings. (**A**) The timeline of the experiment. (**B**–**D**) Changes in body weight, splenic weight and splenic index in control and GoAstV groups at 1, 3, 6, 9 dpi. (**E**). The anatomical diagram of the spleen is at 6 dpi. (**F**) Viral load of spleen. Con (control group); Dis (GoAstV group). All data are expressed as M ± SE and then examined utilizing an independent samples *t*-test. *p* < 0.05 (*). *p* < 0.01 (**). *p* < 0.001 (***).

**Figure 2:**
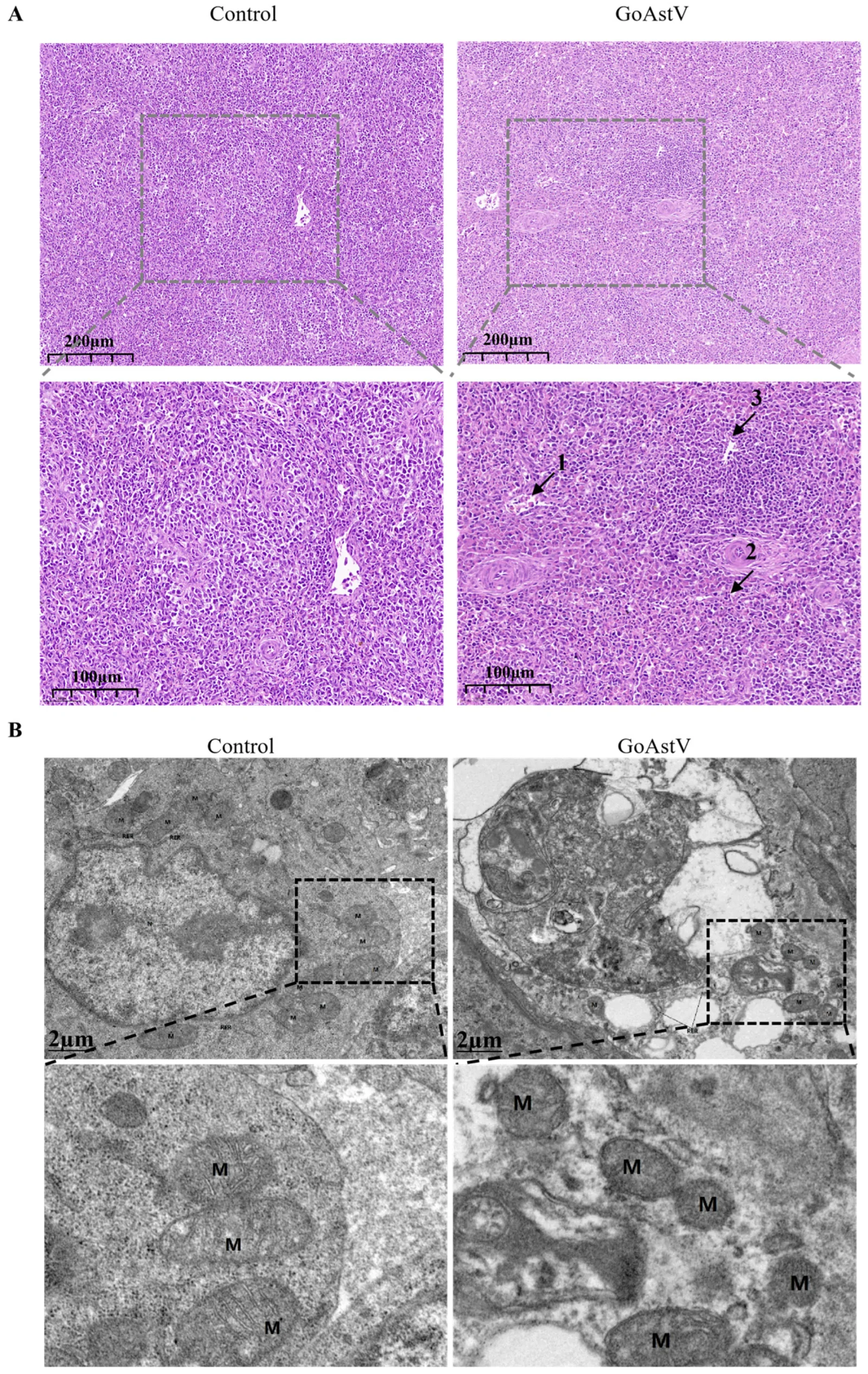
Observation of histopathology and ultrastructure of the spleen. (**A**) Representative H&E-stained sections of splenic tissues of both groups at 6 dpi. 1–3: Lymphocyte necrosis in the spleen; Scale bar: 200 µm (**top**) and 100 µm (**bottom**). (**B**) Observation of the ultrastructure of splenic tissues of both groups at 6 dpi. 1: Abnormal mitochondria. 2: Mitochondrial autophagosome. N: nucleus, M: mitochondria, RER: endoplasmic reticulum; Scale bar: 2 µm. Con (control group); Dis (GoAstV group).

**Figure 3:**
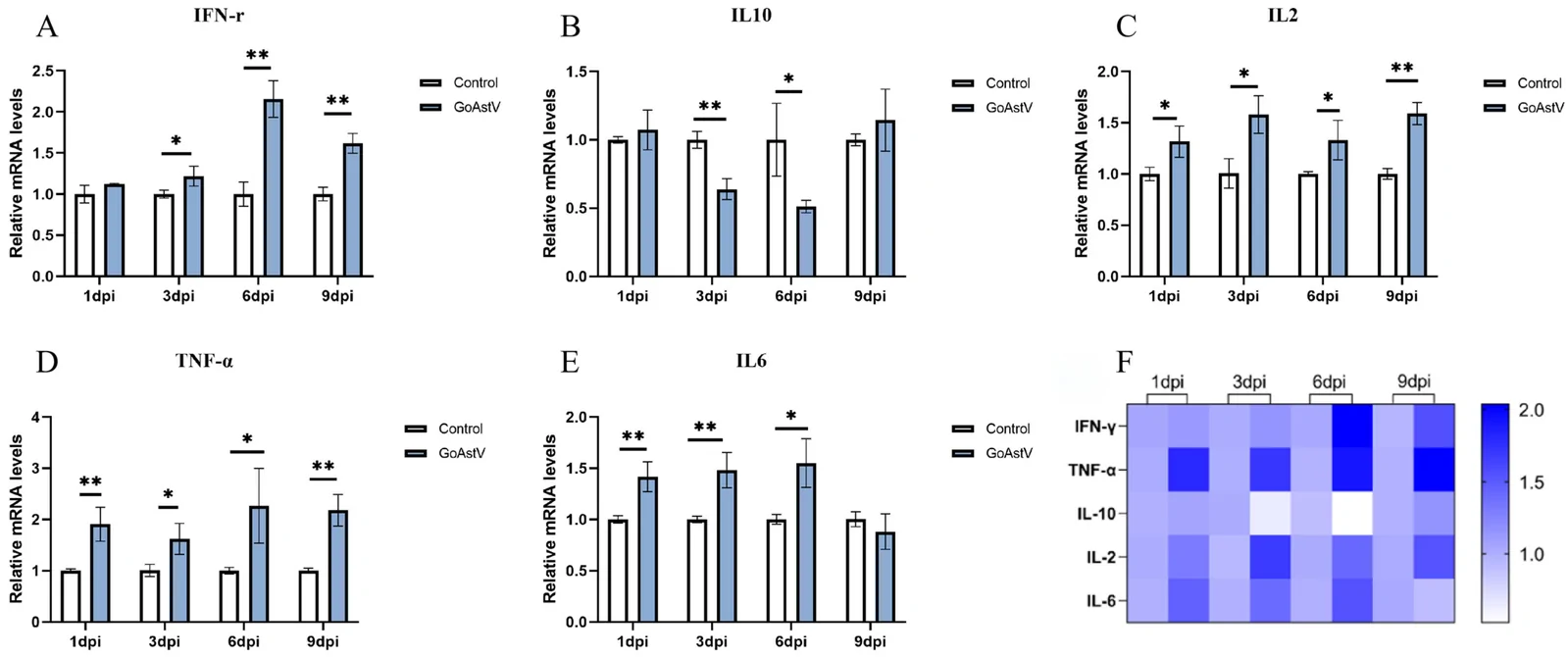
Changes in inflammatory-related molecules in the spleen of GoAst-infected goslings. Expression levels of mRNA of Splenic inflammatory factors IFN-γ (**A**), IL-10 (**B**), IL-2 (**C**), TNF-α (**D**), and IL-6 (**E**). (**F**) The heatmap illustrates the expression levels of genes associated with inflammatory factors. Con (control group); Dis (GoAstV group). All data are expressed as M ± SE and analyzed using independent samples *t*-test. *p* < 0.05 (*). *p* < 0.01 (**).

**Figure 4:**
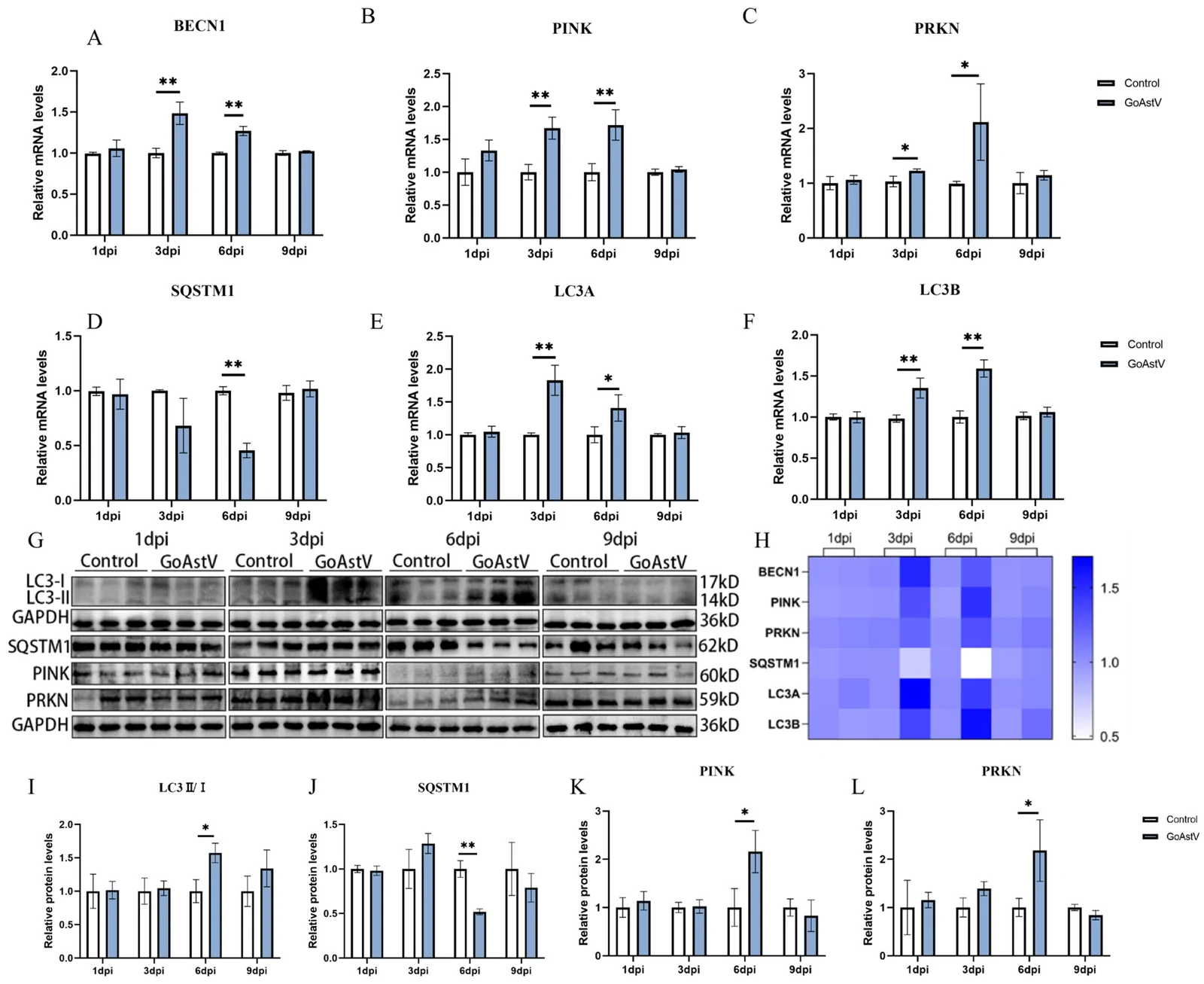
GoAstV-induced mitophagy of spleen cells. Expression levels of mRNA for mitophagy pathway-related genes BECN1 (**A**), PINK (**B**), PRKN (**C**), SQSTM1 (**D**), LC3A (**E**), and LC3B (**F**). (**H**) The heatmap illustrates the expression levels of genes associated with mitophagy channels. (**G**–**L**) Western blot analysis to quantify the relative expression levels of PINK, PRKN, SQSTM1, and LC3II/I proteins. The densitometric values of the various proteins were normalized to GAPDH protein levels. (n = 3) Con (control group); Dis (GoAstV group). All data are presented as M ± SE and evaluated using an independent samples *t*-test. *p* < 0.05 (*). *p* < 0.01 (**).

**Figure 5:**
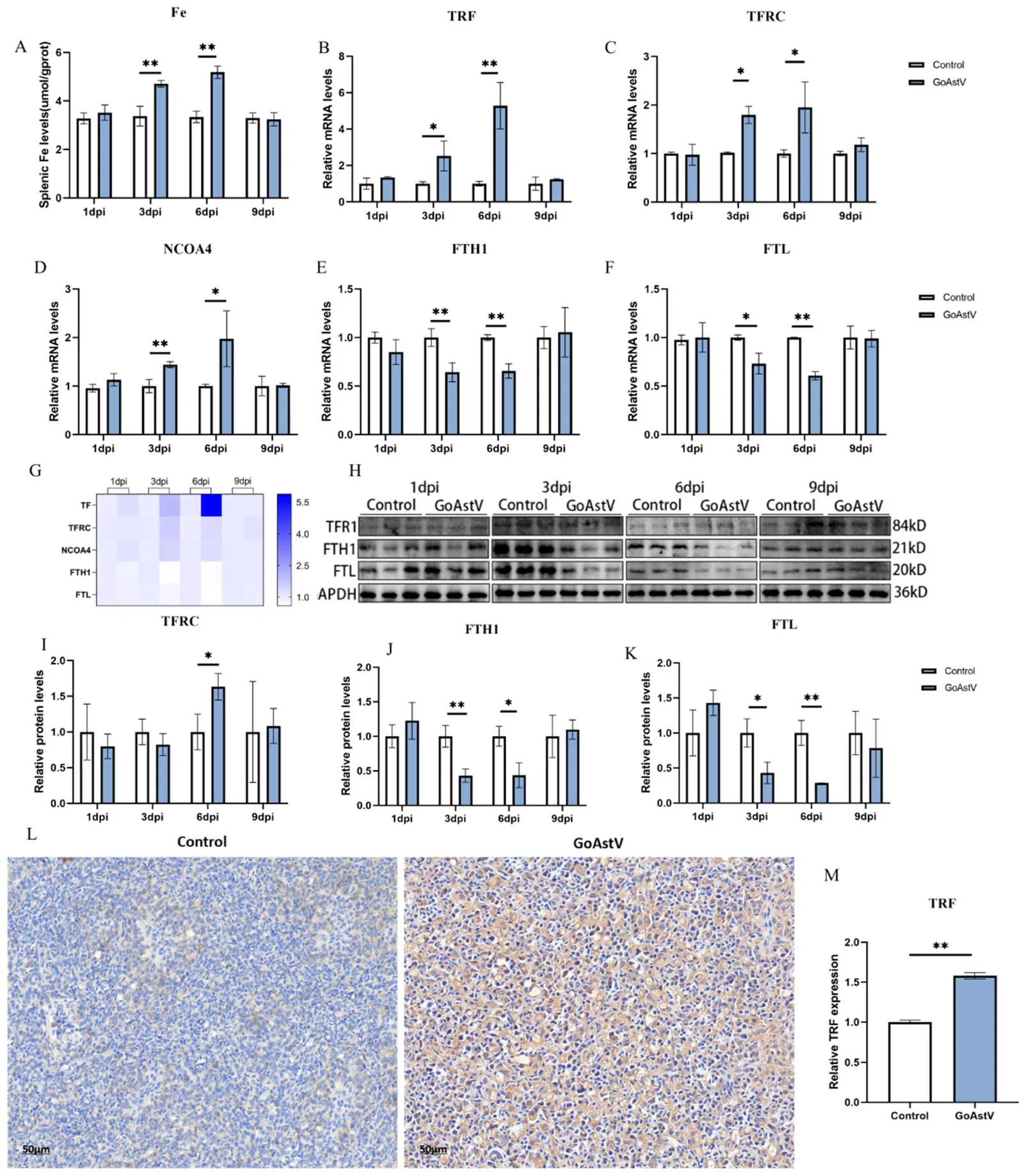
Effect of GoAstV infection on splenic iron metabolic pathway. (**A**) Changes in total iron content in spleen tissues of two groups. Expression levels of mRNA for iron metabolic channel-associated genes TRF (**B**), TFRC (**C**), NCOA4 (**D**), FTH1 (**E**), and FTL (**F**). (**G**) The heatmap illustrates the expression levels of genes associated with iron metabolic channels. (**H**–**K**) Western blot analysis to quantify the relative expression levels of TFRC, FTH1, and FTL proteins. The densitometric values of the various proteins were normalized to GAPDH protein levels. (n = 3) (**L**,**M**) Immunohistochemical staining at 6 dpi and ImageJ analysis of positive expression of TF (Scale bar: 50 µm). Con (control group); Dis (GoAstV group). All data are presented as M ± SE and evaluated using an independent samples *t*-test. *p* < 0.05 (*). *p* < 0.01 (**).

**Figure 6:**
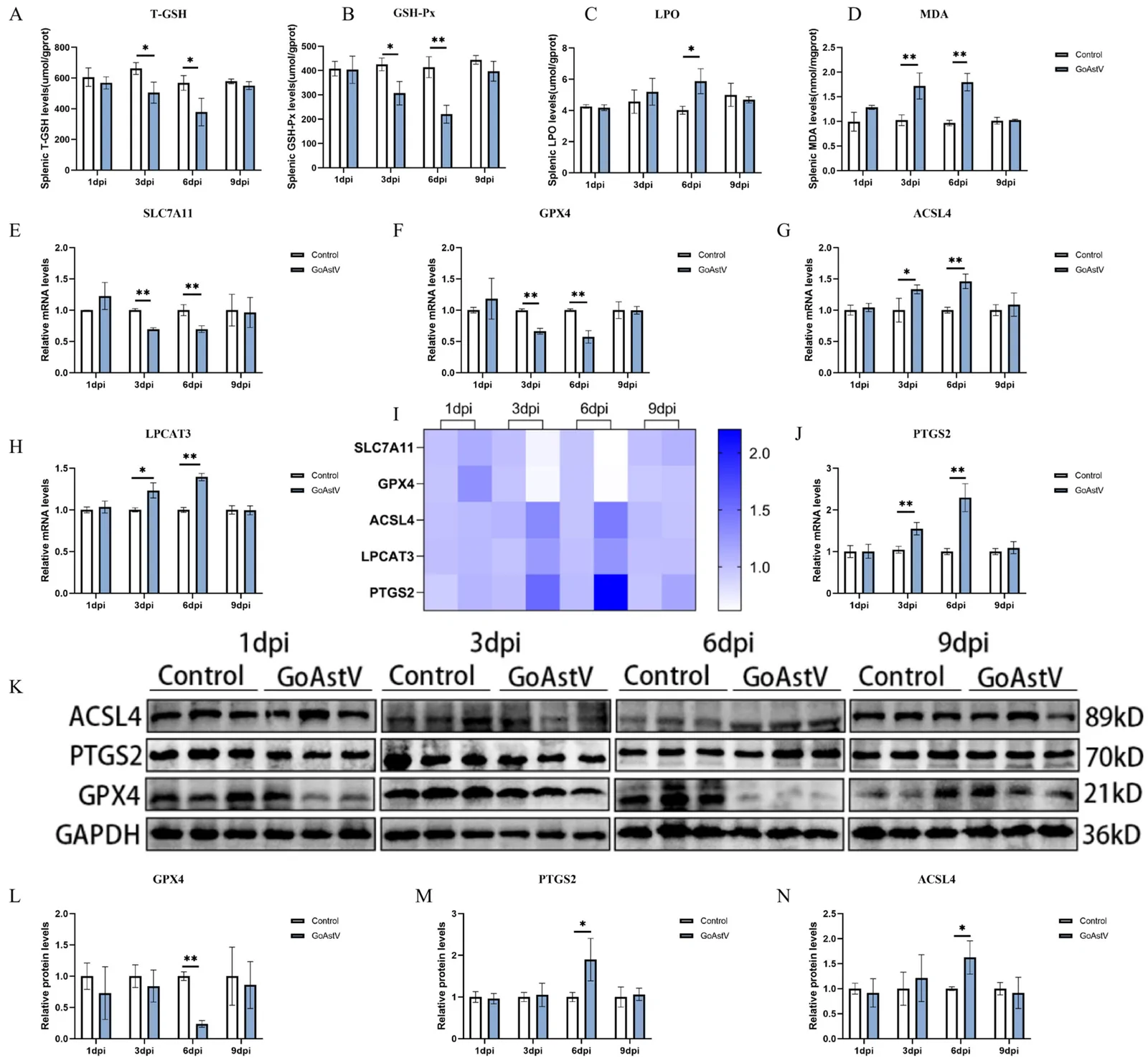
(**A**) T-GSH content (μmol/gprot). (**B**) GSH-Px content (μmol/gprot). (**C**) LPO content (μmol/gprot). (**D**) MDA content (nmol/mgprot). Expression levels of mRNA for GSH synthesis-related genes and lipid peroxidation-related genes SLC7A11 (**E**), GPX4 (**F**), ACSL4 (**G**), LPCAT3 (**H**), PTGS2 (**J**). (**I**) The heatmap illustrates the expression levels of genes associated with GSH synthesis and lipid peroxidation. (**K**–**N**) Western blot analysis to quantify the relative expression levels of GPX4, ACSL4, and PTGS2 proteins. The densitometric values of the various proteins were normalized to GAPDH protein levels. (n = 3) Con (control group); Dis (GoAstV group). All data are presented as M ± SE and evaluated using an independent samples *t*-test. *p* < 0.05 (*). *p* < 0.01 (**).

**Figure 7:**
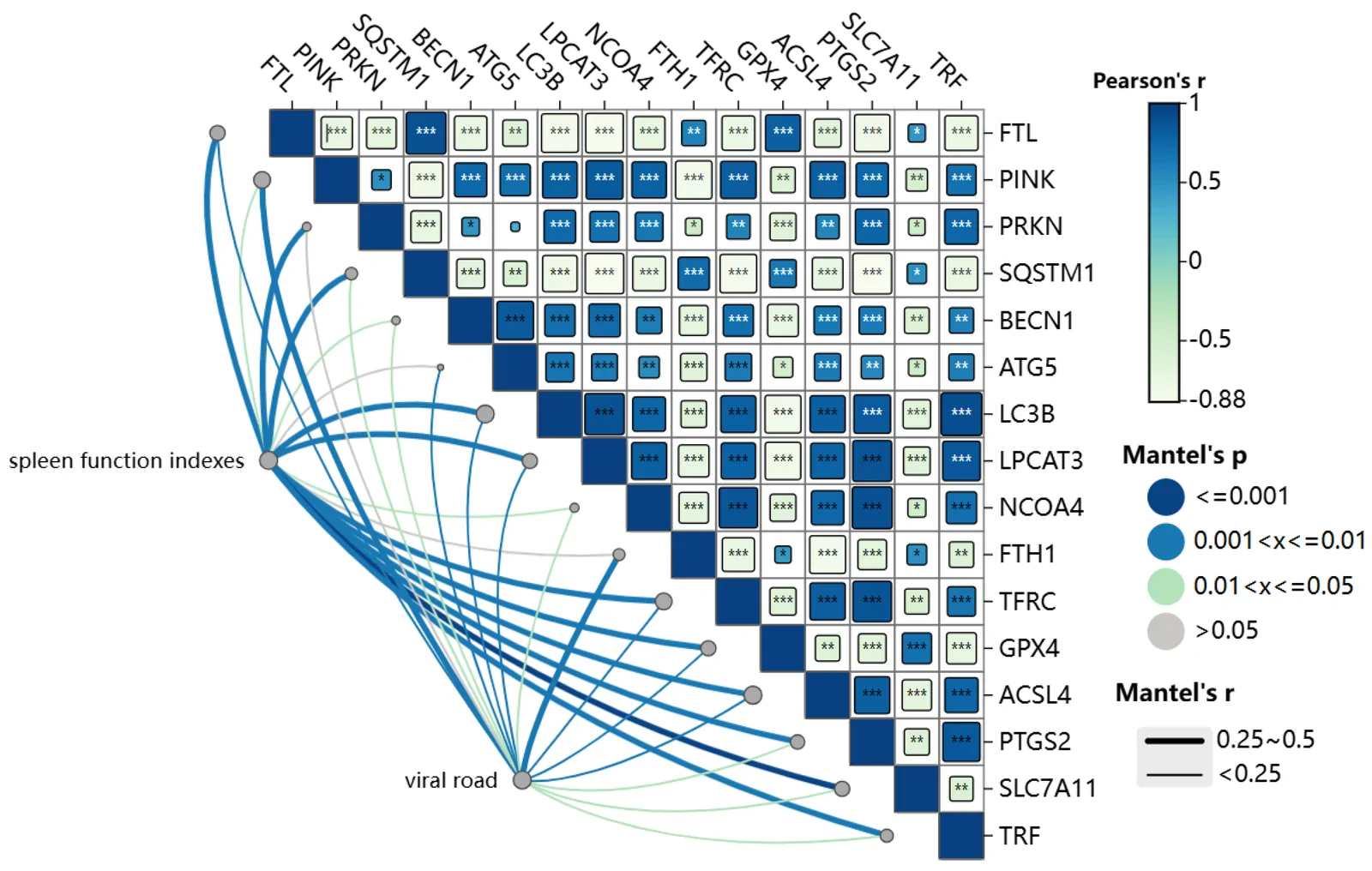
The correlation analysis results of the Mantel test. Mantel’s *p* represents the relationship between spleen function indicators, viral load, and various genes. Pearson’s r represents the correlation coefficient between autophagy- and mitophagy-related genes and ferroptosis-related genes. (drawn on the Chiplot website: https://www.chiplot.online/) *p* < 0.05 (*). *p* < 0.01 (**). *p* < 0.001 (***).

**Table 1:** Primer sequence.

Gene	Accession Number	Primer Sequences (5′ to 3′)
*ACSL4*	XM_048070776.1	F: TTGTTACGTTGTATGCCACCCT
		R: GTTTGGCTCCCAGCTCTTCC
*PTGS2*	XM_048058960.1	F: GATTGACAGCCCACAACTTACA
		R: GATGAGTGAAGTGTTGCAAAG
*GPX4*	XM_066983641.1	F: TTACGCATCCTGGGCTTTCC
		R: GGCTGCTCCTTCATCCACTTC
*SLC7A11*	XM_013189191.3	F: TGGTAGTTGCTGGGTTGA
		R: GACTGGGTCGGAGTAAAG
*TFRC*	XM_067002179.1	F: AGAATGGCTGGAGGGGTACT
		R: TTCTCTCCAGCAGCGCATAC
*FTH1*	XM_066997196.1	F: GAACGACCCGCATCTGTGTG
		R: TGCCATGCCATACTTGGGTG
*NCOA4*	XM_067000715.1	F: AGAGAACTGGCGGGAGGTAA
		R: GCAACGCTTCCTCTTTCAGC
*LPCAT3*	XM_048051525.2	F: GGGTACAATGGCAAGGACCA
		R: ACGCTTGAAGATGTAGCGGG
*FTL*	XM_013177727.3	F: AAATGCAGACCCTCATAGCG
		R: ATGCCTGTCCTGAACTCCTTCT
*PINK*	XM_066982278.1	F: TGTGCTGTAACGCCAAGGG
		R: CGAACACCGTCTGAAAGGAA
*PRKN*	XM_013186798.3	F: CCCAGCAGAGTATCGTTCA
		R: TCCAGAATAACAGCCAACC
*SQSTM1*	XM_048057319.2	F: GCTAAATCCGTTTGAGTGGC
		R: CCAGATTGGGAGAAACAGGAG
*BECN1*	XM_048051865.2	F: GTACCAGAAGGTACTGCGAGTT
		R: TTATTTATCGTGCCGAACTGC
*LC3A*	XM_048048972.2	F: TCGCGGATCGTTGTAAAG
		R: TAGCGTTCAATGATGACTGG
*LC3B*	XM_067004573.1	F: TCCAACCAGGCGTTCTTC
		R: CGCACTCCGAAAGTCTCC
*IFNγ*	XM_013198313.3	F: ATACTGAGCCAGATTGTTTCC
		R: CACGAGGTCTTGAACTTCTTAT
*IL2*	XM_048078148.2	F: ACACCAAATGAGAAACAGGAG
		R: GTCCGTAAGTTTTTGGAGATTC
*IL6*	XM_048070285.2	F: ACGACTAAGGCAGATGGTGATA
		R: TCAAATAGCGAACAGCCCTCA
*IL10*	XM_048071021.2	F: GAACAGCGAGCACCACCAG
		R: TTCCTCCTCTTCATCACAAGTA
*TNFα*	NM_204267.2	F: CACATGGAAGATCACGCAAAG
		R: GTGGTGGGAAGGGAGCAAT
*TF*	XM_013186329.3	F: CAAAACCAAATGCCTCCGCA
		R: CCTCTGGGGCATTTTCTTGAAC
*GAPDH*	NM_067004670.1	F: GTGCTGCCCAGAACATTATCC
		R: AGCAGCAGCCTTCACTACCCT

## Data Availability

The original contributions presented in this study are included in the article. Further inquiries can be directed to the corresponding author.
